# Targeting Gut Microbiota with Probiotics and Phenolic Compounds in the Treatment of Atherosclerosis: A Comprehensive Review

**DOI:** 10.3390/foods13182886

**Published:** 2024-09-12

**Authors:** José Patrocínio Ribeiro Cruz Neto, Micaelle Oliveira de Luna Freire, Deborah Emanuelle de Albuquerque Lemos, Rayanne Maira Felix Ribeiro Alves, Emmily Ferreira de Farias Cardoso, Camille de Moura Balarini, Hatice Duman, Sercan Karav, Evandro Leite de Souza, José Luiz de Brito Alves

**Affiliations:** 1 Department of Nutrition, Health Sciences Center, Campus I—Jd. Cidade Universitária, Federal University of Paraíba, João Pessoa 58051-900, PB, Brazil; patrocinio.neto.eltc@gmail.com (J.P.R.C.N.); micamacario@gmail.com (M.O.d.L.F.); deborah.albuquerque@academico.ufpb.br (D.E.d.A.L.); els@academico.ufpb.br (E.L.d.S.); 2 Department of Physiology and Pathology, Health Sciences Center, Federal University of Paraíba, João Pessoa 58037-760, PB, Brazil; rayanne.mr.alves@gmail.com (R.M.F.R.A.); emmily.farias@gmail.com (E.F.d.F.C.); camille.balarini@gmail.com (C.d.M.B.); 3 Department of Molecular Biology and Genetics, Çanakkale Onsekiz Mart University, Çanakkale 17000, Türkiye; hatice.duman@comu.edu.tr (H.D.); sercankarav@comu.edu.tr (S.K.)

**Keywords:** atherosclerosis, nutraceuticals, gut microbiota, probiotic, quercetin, resveratrol

## Abstract

Atherosclerosis (AS) is a chronic inflammatory vascular disease. Dysregulated lipid metabolism, oxidative stress, and inflammation are the major mechanisms implicated in the development of AS. In addition, evidence suggests that gut dysbiosis plays an important role in atherogenesis, and modulation of the gut microbiota with probiotics and phenolic compounds has emerged as a promising strategy for preventing and treating AS. It has been shown that probiotics and phenolic compounds can improve atherosclerosis-related parameters by improving lipid profile, oxidative stress, and inflammation. In addition, these compounds may modulate the diversity and composition of the gut microbiota and improve atherosclerosis. The studies evaluated in the present review showed that probiotics and phenolic compounds, when consumed individually, improved atherosclerosis by modulating the gut microbiota in various ways, such as decreasing gut permeability, decreasing TMAO and LPS levels, altering alpha and beta diversity, and increasing fecal bile acid loss. However, no study was found that evaluated the combined use of probiotics and phenolic compounds to improve atherosclerosis. The available literature highlights the synergistic potential between phenolic compounds and probiotics to improve their health-promoting properties and functionalities. This review aims to summarize the available evidence on the individual effects of probiotics and phenolic compounds on AS, while providing insights into the potential benefits of nutraceutical approaches using probiotic strains, quercetin, and resveratrol as potential adjuvant therapies for AS treatment through modulation of the gut microbiota.

## 1. Introduction

Cardiovascular disease (CVD) is the leading cause of death worldwide, with atherosclerotic CVD being the most prevalent [1]. Atherosclerosis (AS) is a clinical condition characterized by inflammation associated with lipid accumulation in large arteries [2]. Atherogenesis begins with lesions in the vascular endothelium and progresses to the growth of atherosclerotic plaques that compromise the vessel lumen and eventually rupture, resulting in thrombosis that can lead to stroke or myocardial infarction [3,4].

The influence of gut dysbiosis on the development of clinical disorders such as AS, heart failure, arterial hypertension, and dyslipidemia has been reported [5,6,7]. Evidence suggests that gut dysbiosis is involved in the development of AS through the dysregulation of lipid metabolism, immune-inflammatory responses, and the production of microbiota-derived metabolites that can increase the formation of atherosclerotic plaque [8]. For example, a decrease in the proportion of the Bacteroidetes phylum and an increase in the Firmicutes phylum have been associated with AS progression [6,9].

Trimethylamine N-oxide (TMAO) and lipopolysaccharide (LPS) are pro-atherogenic metabolites produced by the gut microbiota [6,10]. These metabolites are important in the progression of AS and adverse cardiovascular outcomes, since they are associated with atherosclerotic plaque formation, increased production of inflammatory cytokines, and vascular and systemic inflammation [11,12,13,14,15]. Due to the association between gut dysbiosis and the development of CVD, the gut microbiota has recently become a therapeutic target to be explored [16,17].

Phenolic compounds and probiotics have been explored as potential strategies for the prevention and adjuvant treatment of AS due to their cholesterol-lowering, antioxidant, and anti-inflammatory properties [18,19]. These compounds can modulate the composition of the gut microbiota and improve the intestinal barrier function, contributing to improving lipid profile, oxidative stress, and inflammation [20,21,22,23,24,25,26]. Due to these effects, phenolic compounds and probiotics (especially those of the genus *Lactobacillus*) are considered promising alternatives for AS treatment or prevention [27,28,29,30].

The present review aimed to summarize the key findings regarding the use of these individual components in AS, while providing insights into the potential benefits of a nutraceutical approach using probiotic strains and phenolic compounds combined as potential adjuvant therapies for AS treatment through modulation of the gut microbiota.

## 2. Atherosclerosis

Risk factors such as dyslipidemia, oxidative stress, inflammation, arterial hypertension, and diabetes are recognized risk factors for AS and endothelial dysfunction. The increase in endothelial permeability allows low-density lipoprotein (LDL) particles in plasma to penetrate the subendothelial space [31]. The LDL is then oxidized, transforming into oxidized LDL (Ox-LDL) through free radicals and the lipoxygenase pathway. The loss of antioxidants, such as alpha-tocopherol and carotenoids, and the low degradation of polyunsaturated fatty acids (PUFAs), such as arachidonic and linoleic acids, are important factors in LDL oxidation [32].

Ox-LDL is considered an antigenic particle and evokes the expression of chemoattractant proteins and cell adhesion molecules, such as vascular cell adhesion molecule 1 (VCAM-1), endothelium selectins (E-selectins), and platelet selectins (P-selectins), causing migration of leukocytes. Mast cells and T lymphocytes release proinflammatory cytokines and activate the formation of reactive oxygen species (ROS), using pathways that involve nicotinamide adenine dinucleotide phosphate oxidase (NADPH oxidase), xanthine oxidase, uncoupled endothelial NO synthase (eNOS), lipoxygenases, and myeloperoxidase [33,34].

Endothelial cells and Ox-LDL mobilize monocytes into the subendothelial space, which differentiate into macrophages expressing scavenger receptors such as CD36, SRA and LOX-1, and take up Ox-LDL. LOX-1 is a lectin-like receptor that participates in the transcription of nuclear factor kappa β (NF)-κβ. It increases the activity of metalloproteinase (MMP) 1, 2, 3 and 9 (MMP-1, MMP-2, MMP-3 and MMP-9), which contributes to the destabilization of the atherosclerotic plaque [35,36,37,38]. Once macrophages engulf Ox-LDL, its accumulation alters their morphology, transforming them into foam cells. These cells secrete inflammatory cytokines and chemokines such as TNF-α, TGF-β, IL-1β, IL-6, and IL-8, attracting more immune cells in a self-perpetuating cycle [39].

Reactive oxygen species, inflammation, and Ox-LDL stimulate smooth muscle cell migration and collagen deposition, increasing the size of atherosclerotic lesions and compromising blood flow through the vascular lumen. When this process occurs in a coronary artery, blood flow to the heart muscle is limited [32].

The increased activity of MMP can lead to plaque rupture, exposing tissue factors that lead to thrombosis [40]. Recent studies show that gut dysbiosis plays an important role in the development of AS, as well as other diseases such as obesity [41], type 2 diabetes [42], arterial hypertension [43], inflammatory bowel disease [44], and kidney disease [45]. Their relationship to gut dysbiosis will be explored in the Section 3.

## 3. Gut Dysbiosis and Atherosclerosis

The human gut microbiota is a complex and dynamic ecosystem of microorganisms, including archaea, fungi, protozoa, viruses, and bacteria [46]. Food metabolism, vitamin synthesis, fermentation of indigestible food, drug metabolism, formation of the gut epithelial barrier, and antagonism of pathogenic bacteria are some of the physiological functions of the gut microbiota [47,48]. Furthermore, the gut microbiota is involved in the regulation of host metabolic processes such as glucose metabolism, protein–lipid metabolism [49], energy homeostasis, regulating appetite, and influencing the development and function of adipose tissue [50].

Alpha (α), and beta (β) diversity are parameters used to assess the heterogeneity of the gut microbiota. Alpha diversity is the observed richness (number of taxonomic groups), evenness (relative abundance of those taxa), or both in a single sample. Chao1, Simpson’s, and Shannon are some indices used in alpha diversity. Beta diversity is a parameter used to measure the similarity or dissimilarity in samples of two or more communities. Bray–Curtis dissimilarity and Jaccard are the most used beta metrics [51,52,53,54]. Studies have shown that mice with AS present low alpha diversity compared to controls [55].

Under healthy conditions, the gut microbiota is characterized by high taxonomic diversity, stable core microbiome composition, and high microbial genetic richness. Firmicutes and Bacteroidetes comprise approximately 90% of gut microbiota in humans at the taxonomic level, as well as Proteobacteria, Fusobacteria, Tenericutes, Actinobacteria, and Verrucomicrobia [56].

Short-chain fatty acids (SCFAs) are produced in the intestine through the fermentation of partially or non-digestible polysaccharides [57]. These molecules maintain intestinal homeostasis through cellular proliferation and differentiation [58]. The main source of SCFAs is microbial fermentation in the jejunum, ileum, proximal colon, descending colon, sigmoid, and rectum. Acetate, propionate, and butyrate are the main types of SCFA. They represent about 95% of SCFAs in the gut, with an estimated ratio of 3:1:1 [57].

The healthy intestinal barrier consists of three interdependent layers that provide a physical barrier against bacterial intrusion from the intestinal lumen. These are the luminal mucus layer, the gut epithelial layer, which presents a continuous layer of epithelial cells, and the inner layer that forms the mucosal immune system. This barrier is selectively permeable, allowing the translocation of essential dietary nutrients, amino acids, sugars, water, electrolytes, SCFAs, and some microbial metabolites from the intestinal lumen into the circulation [59].

Tight junctions (TJs) are important in maintaining the intestinal barrier’s integrity and impermeability, preventing the translocation of both commensal and pathogenic microorganisms [60]. They are characterized as a network of transmembrane proteins that interact and connect laterally adjacent cells near the apical surface of the epithelium [61]. The imbalance between the composition and function of the gut microbiota, and its metabolites, such as TMAO and trimethylamine (TMA), is known as gut dysbiosis [62,63]. These changes in gut microbiota led to gut bacterial DNA translocation, influencing endotoxins and metabolites absorbed into circulation [64].

It was shown that atherosclerotic mice (CTRP9-KO) had low alpha diversity, a high abundance of Bacteroidetes, and a low abundance of Firmicutes compared to a control group [65]. In addition, it was shown that CTRP9 KO mice receiving fecal microbiota transplantation (FMT) from healthy mice had an increase in the relative abundance of Firmicutes and Ruminococcaceae. On the other hand, healthy mice that received FMT from CTRP9 KO animals had a decrease in Firmicutes and Ruminococcaceae and an increase in Bacteroidetes [65].

An increase in TMAO levels and a high abundance of *Enterorhabdus*, *Romboutsia*, *Proteus*, *Eubacterium nodatum* group, *Escherichia*-*Shigella*, *Eubacterium coprostanoligenes* group, *Parasutterella*, *Muribaculum*, and *Enterococcus* have been reported in AS [66,67]. High TMAO levels are associated with foam cells [68] and activation of the inflammatory response in AS [69].

Another component that has a great impact on AS is LPS. The LPS is a molecule present on the membrane of Gram-negative bacteria and is composed of a lipid A portion, a core oligosaccharide, and the O-antigenic polysaccharide [64,70]. After food intake, LPS levels increase in peripheral circulation and are transported to the liver, where it is degraded by specific liver enzymes or undergoes excretion into the bile via scavenger receptors [70].

**Figure 1:**
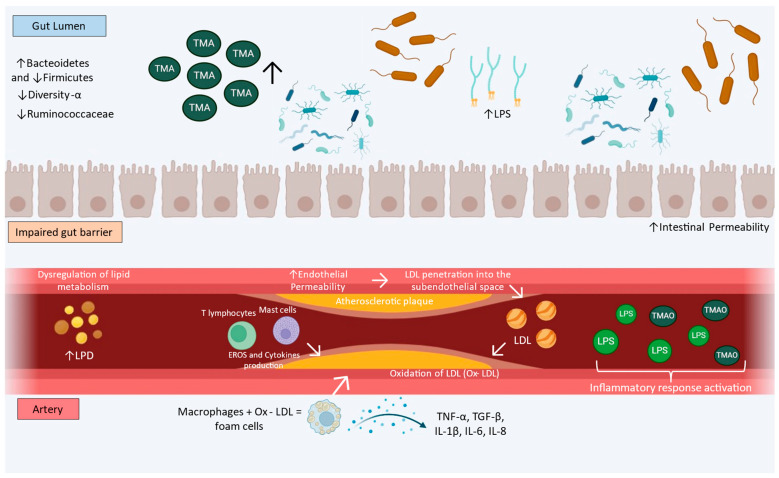
Schematic drawing showing the physiopathology of gut microbiota promoting atherosclerotic plaque formation: ↑ represents an increase and ↓ represents a decrease. TMA: trimethylamine. LPS: lipopolysaccharide. Ox-LDL: oxidized low-density lipoprotein. LDL: low-density lipoprotein. LPD: lipid. TMAO: trimethylamine N-oxide. EROS: reactive oxygen species. TNF-α: tumor necrosis factor-alfa. TGF-β: transforming growth factor beta. IL-1β: interleukin 1 beta. IL-6: interleukin 6. IL-8: interleukin 8.

The LPS induces innate immune system activation by stimulating macrophages and endothelial cells to produce cytokines and inflammatory mediators. The non-degradation or non-excretion of LPS in the bile increases LPS serum levels, promoting low-grade inflammation and a pro-inflammatory process due to the binding to Toll-like receptor 4 (TRL4) [71]. Taken together, these findings indicate that gut microbiota dysbiosis may be involved in the development of AS. Thus, strategies targeting the gut microbiota may represent a therapeutic option for AS. Figure 1 shows the physiopathology of gut microbiota promoting AS.

## 4. Probiotics and Atherosclerosis

“Probiotics are live microorganisms that, when administered in adequate quantities, confer health benefits on the host” [72]. The most used probiotics in the food industry come from fermentative bacteria, such as *Lactobacillus* (L.), *Bifidobacterium* (B.), *Lactococcus*, *Streptococcus*, and *Enterococcus*. Some foods, such as yogurt, kefir, kimchi, and sauerkraut, are sources of probiotic strains [73]. Probiotic strains are important for pathogen antagonism, SCFAs, and vitamin synthesis, maintenance of the epithelial barrier of enterocytes, and modulation of host immunity. It has been shown that probiotics can be used to prevent and treat AS, mainly by improving the gut microbiota, lipid profile, endothelial function, inflammatory profile, and gut microbiota [24,74].

### 4.1. Effects of Probiotics on Gut Microbiome in Atherosclerosis

The role of probiotic treatment of AS has been amply discussed, and gut microbiota modulation is one of the most relevant aspects [24,74]. *Lactobacilli* and *Bifidobacterium* are responsible for reducing the risk of atherosclerosis by breaking down or altering cholesterol metabolism and by reducing inflammation, oxidative stress, and TMAO [75]. Although the authors focused on the cholesterol-lowering effects of these genera, studies show improvement in AS through modulation of the gut microbiota promoted by the administration of strains from these genera [76,77].

A previous study showed that administration of *Lactiplantibacillus* (L.) *plantarum* ATCC 14917 at a daily dose of 10^9^ CFU for 12 weeks to apolipoprotein E knockout (ApoE^−/−^) mice prevented atherosclerotic plaque formation by improving intestinal integrity [78]. The probiotic intervention increased ZO-1, occludin, claudin 3, and MUC-3 mRNA expression in the gut, while decreasing the LPS level in mesenteric adipose tissue [78]. Additionally, another study demonstrated that the administration of a probiotic mixture composed of *B. breve*, *B. longum*, *B. infantis*, *L. acidophilus*, *L. platarum*, *Lacticaseibacillus* (L.) *paracasei*, *L. bulgaricus*, and *Streptococcus thermophilus* for 12 weeks at a daily dose of 2.78 × 10^11^ CFU in ApoE^−/−^ female mice resulted in a distinct compositional profile of the gut microbiota in the ileum and colon when compared with a control group and reduced biomarkers of vascular inflammation and development of AS [79].

Aiming to evaluate the one-to-one effects of five probiotic strains on atherosclerosis treatment, a study tested whether these strains could reduce cecal TMA or serum TMAO levels and TMAO-induced atherosclerosis in mice. Female BALB/c mice fed a chow diet with 1.3% choline chloride were treated with *L. plantarum* ZDY01, *L. rhamnosus* ZDY9, *L. plantarum* ZDY04, *L. casei* ZDY8, *L. bulgaricus* ZDY5 at a dosage of 1 × 10^9^ CFU once a day for four weeks. It was observed that only *L. plantarum* ZDY01 administration reduced serum TMAO and cecal TMA levels. Subsequently, it was reported that *L. plantarum* ZDY04 did not significantly increase the cecal microbiota richness and diversity. However, the administration of *L. plantarum* ZDY04 increased the relative abundance of families such as *Lachnospiraceae*, *Erysipelotrichaceae*, and *Bacteroidaceae*, while decreasing the relative abundance of *Aerococcaceae*. At the genus level, the administration of *L. plantarum* ZDY04 increased the relative abundance of *Enterorhabdus*, *Succinivibrionaceae* UGG-002, *Lachnospiraceae* UGG-006, *Lachnospiraceae* NK4A136, *Ruminiclostridium* 9, *Lachnospiraceae* XPB1014, *Ruminococcaceae* UCG-014, *Ruminococcaceae* NK4A214, *Christensenellaceae* R-7, and *Rikenellaceae* RC9. Lastly, β-diversity measurement revealed that mice treated with *L. plantarum* ZDY04 presented a distinct microbiota structure [80].

Another study evaluated the role of *Limosilactobacillus* (L.) *mucosae* A1 as a potential probiotic in treating atherosclerosis. ApoE^−/−^ mice fed a high-fat, high-cholesterol Western diet were treated with *L. mucosae* A1 (1 × 10^9^ CFU once a day) for 13 weeks. The treatment did not decrease serum TMA, TMAO, or lipopolysaccharide-binding protein (LBP) levels oor the abundance and α-diversity of the gut microbiota, but β-diversity revealed a significant separation of microbial structure between the treated and untreated groups. In the study, it was observed that the treatment with *L. mucosae* A1 decreased the abundance of some all-amplicon sequence variants (ASVs) that were positively correlated with disease phenotypes, which included bacteria belonging to *Oscillibacter*, *Ruminiclostridium*, *Harryflintia*, *Enterorhabdus*, *Anaerovorax*, *Eubacterium*, *Turicibacter*, *Enterococcus*, unclassified *Ruminococcaceae*, unclassified *Clostridiales*, and unclassified *Lachnospiraceae* [81].

A study evaluated the individual anti-atherogenic effects of five probiotic strains, namely, *L. plantarum*, *L. reuteri*, *L. casei*, *B. breve*, and *B. adolescentis*, through intestinal flora and intestinal metabolites in mice fed a Paigen atherogenic diet. For that, female C57BL/6 mice fed a Paigen atherogenic diet were treated with each probiotic at a dosage of 1 × 10^9^ CFU for 16 weeks. Among these probiotic strains, *L. reuteri*, *B. breve*, and *B. adolescentis* significantly increased the fecal concentration of acetate, propionate, and butyrate. Although probiotic treatment did not improve α-diversity, the β-diversity analysis showed a significant difference in the microbiota of treated mice compared to the control group. At the genus level, the treatment with these strains shifted differently the relative abundance of some genera. Firstly, the administration of *B. adolescentis* increased the relative abundance of *Clostridium sensu stricto* 1, *Bifidobacterium*, and *Dubosiella*, and the administration of *L. reuteri* increased the relative abundance of the *Lachnospiraceae* NK4A136 group, *Lactobacillus*, and *Dubosiella*. These genera were positively correlated with fecal acetate and plasma high-density lipoprotein (HDL) concentrations, and negatively correlated with hepatic total cholesterol (TC) and plasma TMAO concentrations. In addition, the administration of *L. plantarum*, *L. reuteri*, and *B. breve* decreased the relative abundance of *Erysipelatoclostridium* and *Romboutsia*. These genera were negatively correlated with fecal acetate concentrations, hepatic superoxide dismutase (SOD) activity, and plasma HDL concentration. Finally, the administration of *L. reuteri* decreased the relative abundance of *Enterorhabdus*. This genus was positively correlated with hepatic TC and plasma TMAO levels and negatively correlated with plasma HDL concentration [77].

ApoE^−/−^ mice were fed a high-fat diet with an additional *L. rhamnosus* GG suspension at a daily dose of 1 × 10^7^ CFU for 12 weeks. It was reported that no significant differences were observed in α-diversity according to Chao 1 and Ace indices, indicating that *L. rhamnosus* GG could not change the total number of intestinal microbial species in these mice. However, the α-diversity was significantly higher according to Simpson and Shannon indices. At the phylum level, *L. rhamnosus* GG consumption decreased the relative abundance of Proteobacteria and increased Firmicutes. Additionally, at the genus level, *L. rhamnosus* GG consumption increased the abundance of *Lactobacillus* while decreasing the abundance of *Desulfovibrionaceae*. β-diversity assessment showed a significant difference in microbial diversity between the group fed a high-fat diet and receiving *L. rhamnosus* GG and the group fed only the high-fat diet. It was also demonstrated that the gut microbiota of the group that received *L. rhamnosus* GG was enriched with *Bilophila* and *Alistipes*, while the microbiota of the group that received only a high-fat diet was enriched with *Deltaproteobacteria* and *Romboutsia* [82]. The main outcomes of probiotic consumption on gut microbiota and AS are summarized in Table 1.

### 4.2. Effects of Probiotics on Lipid Profile Aortic Plaque Deposition in Atherosclerosis

Lowering plasma cholesterol is a common finding after treatment with probiotics, especially when using the genera *Lactobacillus* and *Bifidobacterium* [75]. Administration of a multi-strain probiotic containing *Lactobacillus* and *Bifidobacterium* for 2 weeks reduced plasma cholesterol by 14%, but did not affect LDL, HDL, or triglyceride levels in C57BL/6J mice [83]. Similarly, daily administration of *Lactiplantibacillus* (L.) *pentosus* KF923750 (10^9^ CFU/mL) for eight weeks reduced total cholesterol, triglycerides, and LDL, but did not change the HDL levels in rabbits fed a high-fat diet [84]. Administration of *L. rhamnosus* JL1 (1 × 10^9^ CFU/mL) for 10 weeks promoted hypocholesterolemic effects and increased HDL in C57/BL6 mice fed a high-fat diet [85], suggesting that time intervention is relevant for the hypocholesterolemic effects.

Probiotic co-supplementation with pills containing *L. acidophilus*, *L. reuteri*, *L. fermentum*, and *B. bifidum* (2 × 10^9^ CFU/g each) and selenium for 12 weeks was shown to reduce total cholesterol, very low-density lipoprotein (VLDL), and triglycerides in subjects with coronary artery disease [86]. A randomized, double-blind, placebo-controlled trial supports these findings. Thirty patients with coronary artery disease who received probiotics containing strains of *B. bifidum*, *L. casei*, and *L. acidophilus* (2 × 10^9^ CFU/g each) for 12 weeks showed an increase in HDL levels and a decrease in total cholesterol and atherogenic index [87].

Among the different species of Lactobacillus, *L. fermentum* is effective in the treatment of cardiovascular diseases, including AS. Several studies demonstrate that it acts on the three pillars of AS: hypercholesterolemia, endothelial dysfunction, and inflammation [88,89,90,91].

Studies in ApoE^−/−^ knockout animals demonstrated the hypocholesterolemic effect of probiotics. Administration of *L. mucosae* A1 (1 × 10^9^ CFU/mL) for 13 weeks reduced serum and liver lipids in ApoE^−/−^ mice fed a Western diet [81]. In the same model, administration of *L. acidophilus* ATCC 4356 (1 × 10^9^ CFU/mL) for 16 weeks reduced serum cholesterol, LDL, VLDL, and IDL, as well as intestinal cholesterol absorption [29]. Another mouse model of AS is the LDL receptor knockout animal (LDLr^−/−^). It has been shown that administration of a multi-strain probiotic *L. acidophilus*, *B. bifidum*, *B. animalis*, and *L. plantarum* (5 × 10^8^ CFU/mL) for 12 weeks increased serum HDL levels and reduced LDL, cholesterol, and triglyceride levels [92].

Although these promising findings have been demonstrated, other studies in atherosclerotic animal models have failed to demonstrate a reduction in cholesterol levels with probiotics. Although these studies did not show a hypocholesterolemic effect, a reduction in atheromatous plaques in the aortic sinus has been reported after treatment with probiotic bacteria [28,78,79,93]. Daily administration of *L. plantarum* ATCC 14917 (2 × 10^9^ CFU/mL) for 12 weeks reduced aortic plaque deposition in ApoE^−/−^ mice [78]. Similar results were obtained after multi-strain supplementation with *Streptococcus thermophilus*, *B. breve*, *B. longum*, *B. infantis*, *L. acidophilus*, *L. plantarum*, *L. paracasei* and *Lactobacillus delbrueckii*. The administration of this multi-strain probiotic for 12 weeks (2.78 × 10^11^ CFU/mL) reduced atherosclerotic plaque deposition in ApoE^−/−^ mice [79].

### 4.3. Effects of Probiotics on Endothelial Function in Atherosclerosis

Regarding the action of probiotics on endothelial dysfunction, several studies have demonstrated that the administration of these microorganisms increases the bioavailability of nitric oxide (NO), reduces the generation and impact of reactive oxygen species (ROS), and improves endothelial function [82,90,94,95,96]. Treatment with *L. rhamnosus* (1 × 10^7^ CFU/mL) for 12 weeks in ApoE^−/−^ mice attenuated this injury, partially restoring the integrity of the endothelial layer [82]. Treating rats with probiotic *Weizmannia coagulans* JA845 at 10 mL/kg (1.0 × 10^9^ CFU · mL^−1^) for 6 weeks attenuated the formation of atherosclerotic lesions and alleviated vascular endothelial dysfunction in atherosclerosis induced by vitamin D3 and high-fat diet in rats [97]. A randomized, double-blind, placebo-controlled trial showed that consuming 100 g of yogurt containing *Bifidobacterium animalis* subsp. *lactis* LKM512 (1.0 × 108 CFU/g) and 600 mg of arginine once a day for 12 weeks improved endothelial function in healthy subjects [98]. Whether this combination is effective in improving endothelial function in subjects with atherosclerosis remains to be investigated. A study carried out with the administration of *L. plantarum* 299v (20 billion CFU) in humans with coronary artery disease for six weeks resulted in an improvement in endothelial function, being evaluated based on the reactivity of subcutaneous adipose tissue arterioles by video microscopy [95]. A mechanistic overview of the effects of probiotics in endothelial dysfunction can be seen in a previous review [96].

### 4.4. Effects of Probiotics on Oxidative Stress and Inflammation in Atherosclerosis

Oxidative stress can be assessed by measuring antioxidant enzymes, oxidants, and products of lipid peroxidation such as malondialdehyde (MDA). Probiotic treatment in AS may have a protective effect on the formation and effects of ROS. Administration with probiotics containing *L. acidophilus*, *B. bifidum*, *L. reuteri*, and *L. fermentum* in diabetic patients with CAD increased the total antioxidant capacity and the expression of glutathione peroxidase [89,99]. Furthermore, daily treatment with a probiotic mixture containing *L. acidophilus*, *B. bifidum*, *B. animalis*, and *L. plantarum* for 12 weeks reduced ox-LDL in LDLr^−/−^ [92]. In line with this result, administration of *L. plantarum* also reduced the levels of ox-LDL in ApoE^−/−^ mice. Furthermore, indirect verification of oxidative stress through plasma MDA measurement was reduced after probiotic treatment [27]. MDA levels were also reduced after administration of *L. rhamnosus* in ApoE^−/−^ mice [28]. The analysis of ROS in the aortic tissue of ApoE^−/−^ mice was high compared to healthy mice. On the other hand, treatment with kefir for 12 weeks reduced ROS [100]. It has been shown that probiotics can increase antioxidant SOD activity [28,78,100,101]. Daily administration of *L. acidophilus* 10^9^ CFU/mL for 12 weeks increased serum SOD activity, as well as showing a reduction in the oxidizing enzyme glutathione peroxidase [93]. A previous study showed that daily administration of *L. plantarum* ATCC 14917 (2 × 10^9^ CFU/mL) for 12 weeks increased SOD1 and SO2 mRNA expression in the small intestine [78]. Probiotic strains have important antioxidant properties and can act against oxidative stress [102].

The third important pillar of the action of probiotics against AS is the modulation of the immune system. In the context of AS, inflammatory cytokines are exacerbated at all stages of plaque progression, such as IL-1, TNF, MCP-1, and IL-6, as well as some inflammatory markers, such as C-reactive protein. Administration of probiotics containing *L. plantarum* 299v in patients with CAD resulted in a decrease in pro-inflammatory cytokines IL-8, IL-12, IL-1, and TNFα [95]. The same was observed in treatment for two months with *Lacticaseibacillus rhamnosus* G (1.9 × 10^9^ CFU/mL) in patients with the same disease, in which the levels of CRP and LPS were reduced [103]. *L. fermentum* CQPC07 also demonstrated immunogenic action in obese mice submitted to a high-fat diet. There was a reduction in IL-1, IL-6, IFN and TNFα. Furthermore, anti-inflammatory cytokines such as IL-10 and IL-4 were increased, thus reducing the low-grade inflammation associated with hyperlipidemia [99].

Administration of *L. plantarum* ATCC 14917 for 12 weeks (2 × 10^9^ CFU/mL) in ApoE^−/−^ mice reduced the expression of TNFα and IL-1 in the aorta. In addition, it a reduction in the NF-κβ inflammatory signaling pathway was demonstrated [27]. Building upon these findings, another study using the same probiotic strain showed a decrease in MCP-1, F4/80, ICAM-1, and VCAM-1 mRNA expression in the aorta [78]. The NF-κβ signaling pathway is related to the development of AS, as it generates an inflammatory cascade, potentiating oxidative stress. In this context, a study carried out by Chen et al. (2013) showed that treatment with *Lactobacillus acidophilus* for 12 weeks in ApoE^−/−^ mice resulted in the inhibition of the translocation of NF-κβ p65 from the cytoplasm to the cell nucleus, where it would exert a transcriptional effect due to the presence of IκB proteins, which were previously in deficit in ApoE^−/−^ vehicle animals [93]. Regarding this signaling pathway, similar results were observed by Fang et al., (2019) after administering *L. rhamnosus* in ApoE^−/−^ mice fed a high-fat diet [28].

Probiotics also maintain the epithelial barrier of enterocytes, thus preventing the migration of inflammatory compounds such as LPS into the bloodstream. Studies have shown that inducing LPS in animals increases inflammation and atherosclerotic plaques. This molecule binds to the Toll-like receptors of macrophages, inducing the inflammatory cascade that, in a situation of risk factors, can promote the development of AS [73,104]. Treatment for 12 weeks with *L. plantarum* (2 × 10^9^ CFU/mL) in ApoE^−/−^ mice reduced LPS levels in mesenteric adipose tissue, thereby minimizing its intestinal penetration and consequent inflammatory cascade [78]. Still in this context, a pro-atherogenic substance increased in conditions of dysbiosis is TMAO, which in the bloodstream increases the formation of foam cells, as well as the internalization of LDL in the subendothelial matrix [64]. Probiotics improve AS by modulating the production of this compound. Treatment with *L. plantarum* ZDY04 for 4 weeks (1 × 10^9^ CFU/mL) in ApoE^−/−^ mice supplemented with choline, a substrate to produce TMAO, resulted in an improvement in the progression of atherosclerotic plaques and a reduction in serum levels of TMAO. Ramireddy et al., (2021) used *Lactiplantibacillus plantarum* LP1145, *Lactobacillus amylovorus LAM 1345* and *Limosilactobacillus fermentum* LF33 (10^9^ CFU/mL) to treat C57BL/6 mice fed choline for 28 days. It was observed that serum TMAO levels were reduced in the group treated with the three strains, showing the potent synergistic effect of more than one strain [105].

The findings cited above lead us to understand better the relationship between probiotics and AS through gut microbiota modulation, lipid metabolism regulation, improvement in inflammation, and endothelial function. Several bacteria are important in this process; however, those of the *Lactobacillus* genus play roles proven by various research in the field. Additionally, the improvement in atherosclerotic parameters seems related to the treatment period and the number of strains in the solution. According to the findings cited above, we also observed that the treatment periods are around 10 weeks or more, and most studies evaluated the effects of only one probiotic strain. Moreover, we did not find studies evaluating the mutual uses of probiotic strains and phenolic compounds in AS treatment.

It has been pointed out that *L. fermentum* fruit-derived strains are novel probiotic candidates to promote host health benefits and the development of biotherapeutics [106]. These strains are recognized by their cholesterol-lowing effects, antioxidant, and anti-inflammatory properties [102,107,108]. In addition, it was demonstrated that mutual uses of probiotics and phenolic compounds can improve their health-promoting properties and functionalities [109]. Confirming this, Sampaio et al., (2021) developed a potential nutraceutical combining three *L. fermentum* strains, namely, *L. fermentum* 139, *L. fermentum* 263, and *L. fermentum* 296, with quercetin and resveratrol [110]. In vitro assays have demonstrated that this nutraceutical has high counts of *L. fermentum* viable cells after freeze-drying, high antioxidant capacities, high prebiotic index, and the ability to modulate gut microbiota and metabolite profile on human colonic fermentation [26]. In addition, a nutraceutical combining *L. fermentum* strains with quercetin and resveratrol resulted in a large live *L. fermentum* cell subpopulation, high content and potential bioaccessibility of quercetin and resveratrol, and antioxidant capacity after exposure to simulated gastrointestinal conditions [111].

## 5. Role of Quercetin on Atherosclerosis through Gut Microbiota

Polyphenols are secondary metabolites obtained from plants, and are considered the most abundant and widely distributed natural compounds [112]. Dietary polyphenols are important for human health due to their antioxidant and anti-inflammatory properties [113]. Among polyphenols, quercetin (3,3′,4′,5,7-pentahydroxyflvanone) is a flavonol found in citrus fruit, apples, berries, onions, green tea, green leafy vegetables, seeds, nuts, broccoli, olive oil, grapes, and red wine [114]. The reported biological effects of quercetin include antioxidant and anti-inflammatory, antidiabetic, antihypertensive, antiobesity, antihypercholesterolemic, anti-AS, anticancer, and antitumoral activity [115], and its use as a dietary supplement is generally considered safe [116].

Particularly in CDV disease, quercetin has been considered effective in both prevention and treatment strategies [117]. A systematic review showed that quercetin supplementation at different doses and concentrations promotes cardioprotective effects in rodents fed a high-fat diet [22]. The available literature suggests that quercetin improves cardiovascular health through its effects on platelet aggregation, bacterial growth, cholesterol levels, endothelial cell protection, myocardial protection, and blood pressure regulation [118].

A previous study demonstrated that the atheroprotective effects of quercetin may be caused by the downregulation of proprotein convertase subtilisin/kexin type 9 (PCSK9), and upregulation of peroxisome proliferator-activated receptor γ (PPARγ), liver X receptor α (LXRα), and ATP-binding cassette transporter A1 (ABCA1) in the aortic and liver tissue [119]. Furthermore, quercetin can prevent the inflammatory response in AS via phosphatidylinositide 3 kinase PI3K/protein kinase B (AKT) phosphorylation [120]. Quercetin may also act against endothelial senescence through regulation of p53 and mTOR signaling pathways [121]. Although several pathways are involved in the atheroprotective effects of quercetin, the related improvement in atherogenesis through gut microbiota is still poorly explored.

Polyphenols can modulate several gut microbiota metabolites, such as SCFAs, dopamine, TMA, bile acids, and LPS. Specifically, quercetin is metabolized by several microorganisms found in the gut microbiota that produce metabolites as byproducts. This can alter the diversity and richness of the bacterial community and the specific abundance of some microorganisms, promoting or inhibiting their growth [122]. Although the role of quercetin in improving non-alcoholic fatty liver disease [123], alleviating intestinal inflammation [124], improving obesity-related parameters [125], and improving gut dysbiosis in antibiotic-treated mice [126] has already been pointed out, few studies have explored the role played by quercetin in improving AS-related parameters.

The effect of quercetin on the TLR-NF-κβ signaling pathway in cultured endothelial cells and how it regulates Ox-LDL-mediated adhesion molecules on human umbilical vein endothelial cells (HUVECs) was previously investigated [127]. The authors showed that quercetin at 25 μM concentration reduced VCAM-1 and intercellular adhesion molecule 1 (ICAM-1) expression in HUVECs, as well as enhancing ox-LDL. Additionally, quercetin downregulated MCP-1 mRNA expression, alleviated nuclear translocation of the NF-κβ p65 subunit, and reduced protein and mRNA expression of TLR2 and TLR4. Subsequently, the authors evaluated the therapeutic role of quercetin at a dose of 25 mg/kg in rats fed a hypercholesterolemic diet. Quercetin supplementation reduced inflammatory mediators such as COX, 5-LOX, MOP, NOS, CRP, and IL-6 mRNA expression in rats [127]. Although this study did not specifically evaluate the improvement in AS through gut microbiota, it sheds light on the role of quercetin in an inflammatory pathway that can be activated by gut microbiota-derived metabolites such as LPS.

The effect of quercetin on the improvement in AS-related parameters through gut microbiota was investigated in LDL-receptor knockout mice (LDLR^−/−^). After 12 weeks of treatment with oral administration of quercetin at a dose of 100 µg/day, atherosclerotic lesions in the aortic sinus and malondialdehyde levels were reduced in LDLR^−/−^ mice. Furthermore, quercetin administration decreased levels of cholesterol, lysophosphatidic acids, and atherogenic lysophosphatidylcholine, while increasing coprostanol levels in the ileum. Treatment also decreased cecal total bile acids and cecal cholesterol, while increasing cecal coprostanol. Regarding the gut microbiota, quercetin supplementation increased α-diversity and the relative abundance of Akkermansia, Bacteroides, Parabacteroides, and Ruminococcus [128].

A previous study showed that daily treatment with quercetin at a 100 mg/kg/day for 12 weeks decreased triglycerides (TGs), TC, LDL, and TNF-α while increasing plasma HDL levels in ApoE^−/−^ mice fed a high-cholesterol diet. Regarding the analysis of intestinal microbiota, the treatment did not show significant differences at the phylum level. However, at the genus level, there was a significative increase in 11 genera: *Streptophyta*, *Enterobacter*, *Mobilitalea*, *Clostridium*, *Phascolarctobacterium*, *Candidatus Stoquefichus*, *Faecalimonas*, *Faecalibaculum*, *Anaerovibrio*, *Deltaproteobacteria Unclassified*, and *Acutalibacter*. According to the authors, the genera *Phascolarctobacterium* and *Anaerovibrio* can be considered the main microbiota signature of quercetin treatment. Furthermore, the authors selected 32 metabolic signatures as the key pathways in quercetin treatment, and the primary bile acid biosynthesis pathway was selected as the main pathway [129].

To evaluate the effect of quercetin on the progression of AS in ApoE^−/−^ mice, it was shown that consumption of a diet enriched with quercetin (0.1% *w*/*w*) for 16 weeks did not affect the plasma lipid profile in mice fed a diet low in microbiota-accessible carbohydrates (low-MACs) or a standard grain-based diet (high-MACs). However, quercetin supplementation significantly reduced the size of atherosclerotic lesions. In addition, mice fed a high-MAC diet supplemented with quercetin developed atherosclerotic lesions that contained reduced numbers of macrophages and increased levels of collagen, suggesting that quercetin may reduce vascular inflammation and promote atherosclerotic plaque stability. In addition, quercetin supplementation in a high-MAC diet increased the richness of the gut microbiota in ApoE^−/−^ mice, while it did not alter the richness of the gut microbiota in mice fed a low-MAC diet. Quercetin increased microbiota diversity in both low-MAC and high-MAC diets. Furthermore, in mice fed a high-MAC diet, atherosclerotic plaque areas were negatively associated with the Eggerthellaceae and Erysipelotrichaceae families and positively associated with the Lactobacillaceae family. Finally, the authors also showed that metabolites such as benzoylglutamic acid, 3,4-dihydroxybenzoic acid (protocatechuic acid) and its sulfate form, trans-4-hydroxy-3-methoxycinnamic acid (ferulic acid), and 3-methoxybenzoic acid methyl ester were significantly increased in mice consuming a high-MAC diet supplemented with quercetin compared to mice fed a high-MAC diet without quercetin supplementation [130].

In summary, the findings suggest that quercetin plays an important role in the treatment of AS and that the gut microbiota is a target of quercetin to promote its effects on AS. However, none of the studies found quercetin acting on inflammatory/anti-inflammatory pathways such as LPS-TLR4 and TMAO. Furthermore, no studies were found on the use of quercetin in synergy with other phenolic compounds or probiotics to treat AS through gut microbiota.

## 6. Role of Resveratrol in Atherosclerosis and Gut Microbiota

Resveratrol (3,5,4′-trihydroxy-trans-stilbene) is a natural polyphenolic compound of the stilbene class, which has trans and cis isomers, with the trans form being the most abundant [131,132]. Major dietary sources of resveratrol include grapes, red wine, peanuts, berries, dark chocolate, pistachios, and jackfruit [133,134,135], and it can also be found in non-edible plants such as *Polygonum cuspidatum* (Asian grass), pine bark, and lilies [133]. In addition to these natural sources, resveratrol can be chemically synthesized and is considered an increasingly valuable product on the global market [136].

The health benefits of resveratrol began to be initially described with a focus on its cardioprotective activity [137]. Since then, studies on resveratrol have intensified, demonstrating the diverse biological functionalities of this natural bioactive compound, including anti-inflammatory, antioxidant, anticancer, anti-aging, cardioprotective, and neuroprotective effects [133,138,139,140,141,142]. These beneficial effects have been observed in a wide range of clinical conditions, including cardiovascular disease, diabetes, Alzheimer’s disease, some types of cancer, colitis, and kidney disease [43,141,143,144,145,146,147].

A growing body of evidence supports a variety of mechanisms that have been identified to mediate the protective effects of resveratrol against cardiovascular disease, particularly AS [148,149]. Among the beneficial effects on AS, it has been observed that resveratrol can promote a reduction in TC, TG, and LDL and an increase in HDL serum levels [149,150,151], possibly through regulation in hepatic enzyme 3-hydroxy-3-methylglutaryl coenzyme A (HMG-CoA) reductase or cholesterol 7α-hydroxylase (CYP7A1) [152,153]. In addition, the antioxidant property of resveratrol has been associated with the inhibition of Ox-LDL [137].

A study showed that daily treatment with resveratrol (5 mg/kg for 5 weeks) in ApoE^−/−^ mice with AS induced by HFD and LPS promoted a reduction in parameters related to the lipid profile and caused an increase in HDL [149]. In addition, the treatment was effective in improving pathological changes in the coronary wall, as well as reducing the infiltrated lesion in the aorta and the percentage of plaque area [149]. In this study, resveratrol was also shown to inhibit the proliferation and activation of CD4+ T cells in mice and in spleen cells cultured in vitro. CD4+ T cells are among those responsible for the adaptive immune response, and when activated, are responsible for secreting pro-inflammatory cytokines that contribute to the initiation and progression of AS [149].

Several routes have been investigated for the protective effects of resveratrol against AS. In rabbits with HFD-induced AS, it was observed that at the vascular level, resveratrol (80 mg/kg/d for 3 months) was able to reduce the thickness of the intima and the intima/media ratio, as well as the smooth muscle layer [150]. The resveratrol intervention also reduced the levels of TC, LDL-C, and blood lipoprotein-associated phospholipase A2 (Lp-PLA2). Lp-PLA2 is an enzyme of the phospholipase A2 family that can be produced by inflammatory cells in atherosclerotic plaques, including monocytes, macrophages, mast cells, and T cells [150]. The binding of Lp-PLA2 to lipoproteins through apolipoprotein B promotes the hydrolysis of oxidized phospholipids in ox-LDL and induces the production of pro-inflammatory lipid substances, leading to vascular endothelial cell apoptosis, endothelial dysfunction, production of adhesion factors and cytokines, and the development of AS [150,154].

Intervention with resveratrol (200 mg/100 g diet for 8 weeks) also promoted an increase in the expression of eNOS mRNA, an enzyme important for the maintenance of endothelial function in the aorta of hyperlipidemic ApoE^−/−^ mice [155]. In addition, the intervention was able to increase protein kinase A (PKA) activity, improve endothelium-dependent vasorelaxation, and reduce the formation of atherosclerotic lesions [155]. These findings suggest that resveratrol may improve endothelial dysfunction in AS, possibly through eNOS activity and PKA signaling, which increase the bioavailability of nitric oxide (NO) [155]. The anti-inflammatory properties of resveratrol were also observed in a study carried out with rats fed a hypercholesterolemic diet, which showed that treatment with resveratrol (50 mg/kg/day for 5 weeks) promoted a significant reduction in IL-1β, NLRP3 inflammasome, ASC, and caspase 1 [156]. In addition, it was shown that resveratrol may act on inflammation by increasing SIRT1, which inhibits NLRP3 transcription and suppresses the NF-κB pathway [156]. Together, these effects promoted by resveratrol promote the improvement in AS and protect against vascular events such as acute myocardial infarction and heart failure [157].

Despite the multiple physiological functions of resveratrol, its bioavailability is significantly low. It has been observed that after oral administration of 25 mg of resveratrol, only a small amount was detectable in serum [158]. It has been observed that resveratrol concentrations in the intestinal mucosa remain considerably high compared to serum levels, suggesting that the intestine is a route for resveratrol to promote its biological functions in the host [159,160]. It has been shown that a significant fraction of resveratrol is metabolized by the gut microbiota and that the varying proportions of gut bacteria and their levels of metabolic activity may be associated with the various health benefits promoted by resveratrol [161,162,163,164]. The composition of the gut microbiota may influence the metabolism of resveratrol, as resveratrol and its metabolites, such as 4-hydroxyphenylacetic acid, and 3-hydroxyphenylpropitric acid [162], can promote changes in the composition and diversity of the gut microbiota, promoting an increase in the proportions of *Bacteroides*, *Lactobacillus* and *Bifidobacterium*, which are considered beneficial to the host [165,166], suggesting that resveratrol has great potential for use as a prebiotic [135,162].

Evaluating the effects of a choline-rich diet supplemented with resveratrol (0.4%) on TMAO-induced AS in C57BL/6J and ApoE^−/−^ mice [165], was shown that resveratrol supplementation reduced TMAO levels, possibly by decreasing gut microbial TMA production. Analysis of cecal content at the genus level showed that resveratrol supplementation increased the relative abundance of *Bacteroides*, *Akkermansia*, *Lactobacillus*, and *Bifidobacterium*, the latter being considered a bacterium capable of increasing bile salt hydrolase (BSH) activity [165]. The gut microbiota modulation promoted by the resveratrol contributed to an increase in BSH activity and consequently to the production of unconjugated bile acids (BAs) from conjugated BA, increasing fecal BA loss in a gut microbiota-dependent manner [165].

Studies on the effects of resveratrol on AS through modulation of the gut microbiota are still scarce. However, the effects of resveratrol modulating the gut microbiota have been observed in other experimental models, promoting improvements in biomarkers related to AS, suggesting that its use may be an excellent therapeutic strategy for clinical conditions related to gut dysbiosis and alterations in cardiovascular mediators of oxidative and inflammatory stress [151,162,163,167]. Studies in obese mice fed a high-fat diet have demonstrated that resveratrol (300 mg/kg/day for 16 weeks by oral gavage) plays an important role in the remodeling of intestinal dysbiosis [162] associated with the improvement in intestinal oxidative stress through the reduction of MDA and increased activity of antioxidant enzymes such as SOD [162], and a significant decrease in serum glucose levels, lipid profile and LPS [163], as well as serum levels of TNF-α and IL-6, and jejunal levels of IL-6, IL-1β and MCP-1 [163].

Although these findings contribute to new insights into the activities of resveratrol in AS, studies on the effects of resveratrol on AS via modulation of the gut microbiota remain underexplored. To this end, it is important to investigate the impact of resveratrol at the intestinal level, such as changes in the composition of the intestinal microbiota, improvement in the intestinal barrier, and production of metabolites. In addition, it is necessary to investigate how the improvement in intestinal dysbiosis may affect signaling pathways associated with the improvement in parameters related to the development and progression of AS.

## 7. Role of Other Phenolic Compounds on Atherosclerosis and Gut Microbiota

The previous sections of this review comprehensively discussed the role of quercetin and resveratrol in AS treatment through gut microbiota modulation. However, other phenolic compounds such as curcumin, gallic acid, naringin, procyanidin, and geraniin may also be involved in AS treatment through gut microbiota modulation [19,168,169,170,171,172].

### 7.1. Curcumin

Curcumin is a polyphenol with multiple health benefits, such as anti-inflammatory and anti-oxidant, and is used in metabolic syndrome [173]. In addition, a study showed the role of curcumin in the treatment of AS [168]. It was shown that daily treatment with curcumin at 100 mg/kg for two months restored the Firmicute/Bacteroidetes ratio, increased the relative abundance of Verrucomicrobia, *Unspecifield*_S24_7, and *Akkermansia*, while decreased the abundance of *Lactobacillus* in ApoE^−/−^ mice. Finally, curcumin intervention was observed to decrease TMAO plasma levels in ApoE^−/−^ mice [168].

In another study, LDLr^−/−^ mice were treated with curcumin at 100 mg/kg for 16 weeks [174]. It was shown that curcumin treatment reduced intestinal permeability and serum LPS levels. In addition, in vitro analyses showed that treatment with 5 µM curcumin for 48 h prior to stimulation with LPS (1 µg/mL) in Caco 2 cells restored protein expression of ZO-1 and claudin 1 protein expression. [174].

### 7.2. Gallic Acid

Gallic acid is recognized for anti-inflammatory, anti-dengue, anti-platelet, anti-apoptotic, anti-cancer, anti-microbial, and antioxidant properties [175]. A study evaluated the effects of gallic acid consumption on AS treatment through gut microbiota modulation in male and female ApoE^−/−^ mice fed a high-fat diet [169]. ApoE^−/−^ mice were randomized into two groups fed a chow diet and 0.2% gallic acid in drinking water for 2 weeks. Gallic acid treatment reduced Gram-positive/negative bacteria in males, but not in females. In addition, gallic acid treatment did not alter beta or alpha diversity in ApoE^−/−^ mice. Finally, the authors observed that gallic acid treatment reduced atherosclerotic plaque formation in males, but not in females [169]. Therefore, it is suggested that gallic acid partially restores microbiome composition by ameliorating high-fat-induced gut dysbiosis in males, and that the reduction in the incidence and progression of atherosclerosis may be sex-dependent.

### 7.3. Naringin

Naringin is a nutritional flavone glycoside that is effective in the treatment of chronic disorders associated with aging [176]. In addition, a study evaluated the effects of naringin on AS treatment through changes in gut microbiota [170]. Female ApoE^−/−^ fed a high-fat diet were treated with naringin at a dosage of 100 mg/kg/day for 16 weeks. The treatment increased total lipid, total bile acids, and coprostanol fecal excretion. Naringin treatment increased the relative abundance of Firmicutes and decreased Bacteroidetes and Verrucomicrobia. In addition, bile salt hydrolase (*Bacteroides*, *Bifidobacterium*, *Lactococcus*, and *Clostridium* sensu_stricto_1)-producing bacteria were decreased after treatment with naringin. TMA-producing bacteria such as *Streptococcus*, *Desulfovibrio*, *Parasutterella*, and *Bacteroides* tended to decrease after naringin treatment. It was also observed that bile acid biosynthesis tended to be increased by naringin, which probably implicates gut microbiota regulation in cholesterol metabolism, particularly bile acid synthesis [170].

Another study evaluated the effects of four citrus flavanones, namely, naringin, hesperidin, naringenin, and hesperidin, on AS treatment. Female ApoE^−/−^ mice fed a high-fat diet were treated with 100 mg/kg/day of each polyphenol for 16 weeks [177]. It was first observed that naringin showed the most antiatherogenic effect. ApoE^−/−^ mice treated with naringin presented a distinct bacterial community related to the other three flavanone treatments. At the genus level, *Lactobacillus*, *Eubacterium coprostanoligenes*, and *Eubacterium brachy* were increased and *Bacteroides*, *Lactococcus*, and *Clostridium sensu stricto 1* were decreased with naringin [177]. *Lactobacillus*, *Clostridium*, *Enterococcus*, and *Bacteroides* were shown to be bile salt hydrolase-producing bacteria and facilitated the hydrolysis of conjugated bile acids. *Eubacterium coprostanoligenes*, and *Eubacterium brachy* are reportedly 7α-dehydroxylase-producing bacteria that catalyze the transformation of conjugated bile acids into their free forms. Therefore, according to that study, the potential pathway for atherosclerosis alleviation of naringin is via the gut microbiota–liver axis [177].

### 7.4. Procyanidin

Known as the second-most abundant polyphenol found in nature, procyanidin is divided into types A and B [178]. Although the role of B-type procyanidin in preventing atherosclerosis has been shown to be effective, the role of A-type procyanidin in AS is still poorly explored [179]. A study evaluated the role of procyanidin A2 in AS treatment. ApoE^−/−^ males fed a high-fat diet were treated daily with proanthocyanidin A2 (110 mg/kg) for 12 weeks. Procyanidin A2 treatment reduced aortic plaque area and macrophage accumulation. Procyanidin A2 treatment was shown to significantly increase gut bacterial α-diversity and promote a distinct microbiome composition. At the genus level, treatment significantly decreased the Firmicutes/Bacteroidetes ratio and increased the relative abundance of Verrucomicrobia. Additionally, at the genus level, the treatment increased the relative abundance of *Akkermansia*, Prevotellaceae, and *Coriobacteriaceae_*UGG-002. It was also observed that the benefits of procyanidin A2 on AS may be attributed to its microbial-derived metabolites. Four metabolites—HPAA, HPPA, 3,4-dihydroxyphenylpropionic acid, and 3,4-dihydroxyphenylacetic acid—were observed in plasma after treatment with procyanidin A2. Finally, correlation analyses revealed that the concentrations of HPAA and HPPA were positively correlated with *Akkermansia*, *Coriobacteriaceae*_UGG-002, and *unclassified*_f_*Prevotellaceae* at the genus level [171].

### 7.5. Geraniin

First identified in *Geranium thunbergia*, geraniin is a natural polyphenol with various biological activities, such as antioxidant, anti-inflammatory, and antihyperglycemic [180,181,182,183]. The role of geraniin on gut microbiota modulation in AS treatment has been previously evaluated [172]. Geraniin (80 mg/kg/day) was dissolved in drinking water and offered to female ApoE^−/−^ mice fed a high-choline diet (0.08% choline diet and 1% choline diet) for 12 weeks. First, it was observed that geraniin consumption significantly reduced serum TMAO levels. At the phylum level, the gut microbiota of geraniin-treated mice was enriched in Bacteroidetes and Firmicutes, which was nearly identical to that observed in chow-fed animals. At the genus level, increases in *Bacteroides*, *Alistipes*, *Flavonifractor*, *Clostridium XIVb*, *Butyricicoccus*, *Anaeroplasma*, *Clostridium XI*, *Enterorhabdus*, *Erysipeiotrichaceae_incertae_sedis*, *Anaerotruncus*, *Intestinimonas*, *Vampirovibrio*, and *Butyricimonas* were observed, while decreases in *Sphingomonas*, *Rhizobium*, *Stenotrophomonas*, *Burkholdeira*, *Serratia*, *Ruminococcus*, *Blautia*, *Parasutterella*, *Pseudomonas*, *Roseburia*, and *Streptophyta* were observed. Finally, it was demonstrated that treatment with geraniin was able to significantly decrease atherosclerotic plaque area [172].

### 7.6. Protocatechuic Acid

Protocatechuic acid is a water-soluble phenolic acid that possesses cardioprotective activity [184,185]. The role of protocatechuic acid in AS treatment through gut microbiota was previously evaluated [186]. Male ApoE^−/−^ mice fed a Western diet supplemented with TMAO were treated for 12 weeks with a Western diet supplemented with protocatechuic acid (0.5% or 1.0%). It was first reported that protocatechuic acid diet supplementation did not decrease TMAO plasma levels. On the other hand, protocatechuic acid diet supplementation improved the inflammatory profile, decreasing TNF-α, MCP-1, IL-1β, and IL-6 plasma levels [186]. Treatment with protocatechuic acid increased α-diversity and caused a cluster to shift closer to the control group in β-diversity. In addition, protocatechuic acid did not change the Firmicutes/Bacteroidetes ratio. The authors also reported that *Mucispirullum*, *Parvibacter*, *Ruminicoccus2*, *Roseburia*, *Enterorhabdus,* and *Odoribacter* were positively associated with inflammatory cytokines, while *Akkermansia*, *Rikenella*, and *Allobaculum* were negatively associated with inflammatory cytokines. Finally, the treatment significantly decreased the aortic plaque area [186]. Table 2 summarizes the use of phenolic compounds in atherosclerosis treatment through gut microbiota changes.

**Figure 2:**
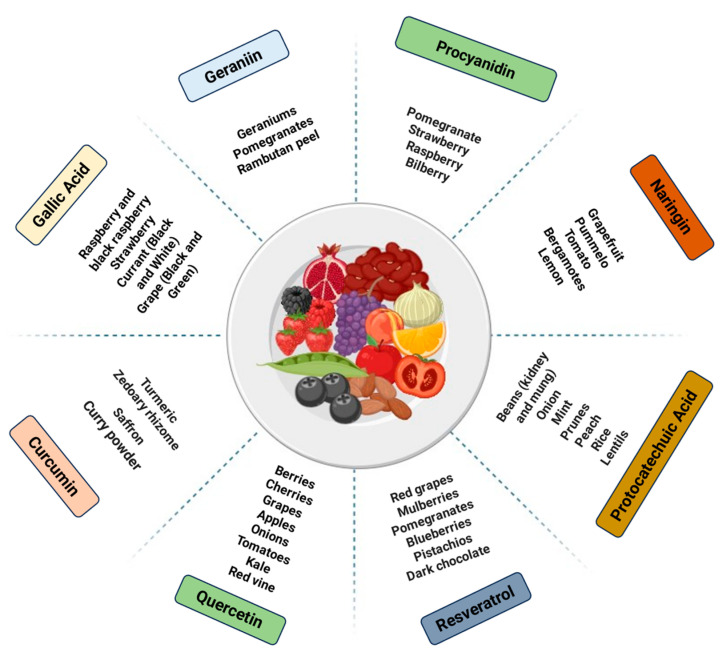
Schematic summarizing the main sources of quercetin, resveratrol, protocatechuic acid, naringin, procyanidin, geraniin, gallic acid, and curcumin.

Taken together, the findings suggest that phenolic compounds may act in the treatment of AS through modulation of the gut microbiota. In this way, it is reasonable to emphasize that a daily dietary intake of foods rich in phenolic compounds may play an important role in preventing or supporting the treatment of AS. Figure 2 summarizes the main sources of these compounds.

## 8. Combined Uses of Probiotics, Quercetin, and Resveratrol as Nutraceutical Candidates for Treating Atherosclerosis

The term “nutraceutical” was first defined by Stephen DeFelice, arising from two broad terms “nutrition” and “pharmaceutical”. According to Hoti et al. (2022), nutraceuticals are bioactive compounds or natural chemicals that present valuable biological activities and have demonstrated physiological benefits. They also work to promote natural healing, prevention, and treatment of diseases. Nutraceuticals are generally phenolic substances, fat acids and structural lipids, carbohydrates and derivatives, amino acid-based substances, minerals, probiotic microorganisms, and isoprenoid derivatives [187].

Nutraceuticals are key players in the treatment of diseases such as metabolic and cardiometabolic disorders. Their bioactive compounds have nutritional and pharmacological properties that attenuate oxidative stress and inflammation [188]. It has been pointed out that nutraceuticals act in both the prevention and treatment of AS [189,190]. Increasing evidence shows that nutraceuticals may play an important role in the treatment of AS, exerting cardiovascular protective effects and reducing an individual’s risk of cardiovascular events such as myocardial infarction and stroke [191,192].

It has been demonstrated that natural products, such as nutraceuticals, can slow the progression of AS by modulating the composition and metabolism of the gut microbiota, inhibiting the migration of monocytes and macrophages, promoting the polarization of the M2 phenotype of macrophages, negatively regulating the levels of inflammatory factors, regulating Treg/Th17 balance, and inhibiting the formation of foam cells [193].

The available literature suggests that the digestion and absorption of dietary polyphenols is poor, with only 5–10% of total ingested polyphenols estimated to be absorbed in the small intestine [194]. Therefore, unabsorbed dietary polyphenols enter the colon, where they are bio-transformed by the resident microbiota, improving their absorption and bioavailability [109,195]. Phenolic compounds can modulate the composition of gut microbiota, mainly through the inhibition of pathogenic bacteria and stimulation of beneficial bacteria. On the other hand, phenolic compounds can exert a prebiotic function and increase the population of beneficial bacteria, including probiotics [196,197,198]. Together, these findings suggest a mutual relationship between phenolic compounds and probiotics, which should enhance their health-promoting properties and functionalities [109].

Based on the studies mentioned above and the absence of evidence regarding the influence of quercetin and resveratrol on the growth and probiotic-related in vitro properties of *Limosilactobacillus fermentum*, a previous study demonstrated that quercetin (2048 µg/mL) and resveratrol (1400 µg/mL) had a weak inhibitory effect on several strains of *Limosilactobacillus fermentum* and did not affect their tolerance to acid pH and bile salts. Furthermore, quercetin and resveratrol improved the ability of *Limosilactobacillus fermentum* strains to self-aggregate and co-aggregate with pathogens, in addition to not affecting the antagonistic activity against pathogens and increasing their survival rates when challenged with simulated gastrointestinal conditions [199].

Considering the positive effects of quercetin and resveratrol on *Limosilactobacillus* strains, a potential nutraceutical was developed in a subsequent study by combining L. fermentum strains, namely, *L. fermentum* 139, *L. fermentum* 263, and *L. fermentum* 296 (109 CFU/mL), and quercetin (500 mg) and resveratrol (300 mg) [110]. The nutraceutical was shown to contain a high number of viable *L. fermentum* cells (±9 log CFU/g) after lyophilization. In addition, the authors showed that the nutraceutical had a high antioxidant capacity when exposed to simulated gastrointestinal conditions, as well as good potential bioaccessibility for quercetin and resveratrol [110]. Nutraceutical stability was evaluated and revealed that the nutraceutical showed high counts of viable *L. fermentum* cells and high levels of quercetin and resveratrol after 30 and 90 days of room storage and refrigeration [110], in addition to presenting high antioxidant capacities during long-term storage under refrigeration or at room temperature [200].

Another study carried out by Sampaio et al. (2022) demonstrated that the nutraceutical increased the relative abundance of *Lactobacillus* spp./*Enterococcus* spp. and *Bifidobacterium* spp., while decreasing the relative abundance of *Bacteroides/Prevotella*, *Clostridium histolyticum*, and *Eubacterium rectale*/*Clostridium coccoides* during colonic fermentation, with a high prebiotic index. Furthermore, the nutraceutical induced the production of lactic, propionic, and butyric acids and increased antioxidant capacity in a medium that mimicked the colonic environment. After 48 h of colonic fermentation, the metabolites citrate, succinate, lactate, formic acid, 5-aminosalicylate, and *N*-acetyl-5-aminosalicylate were detected in media with *L. fermentum*, quercetin, and resveratrol [26]. Finally, the medium containing the nutraceutical tested showed a high amount of subpopulation of live *L. fermentum* cells, high content and potential bioaccessibility of quercetin and resveratrol, and antioxidant capacity after exposure to simulated gastrointestinal conditions [111].

**Figure 3:**
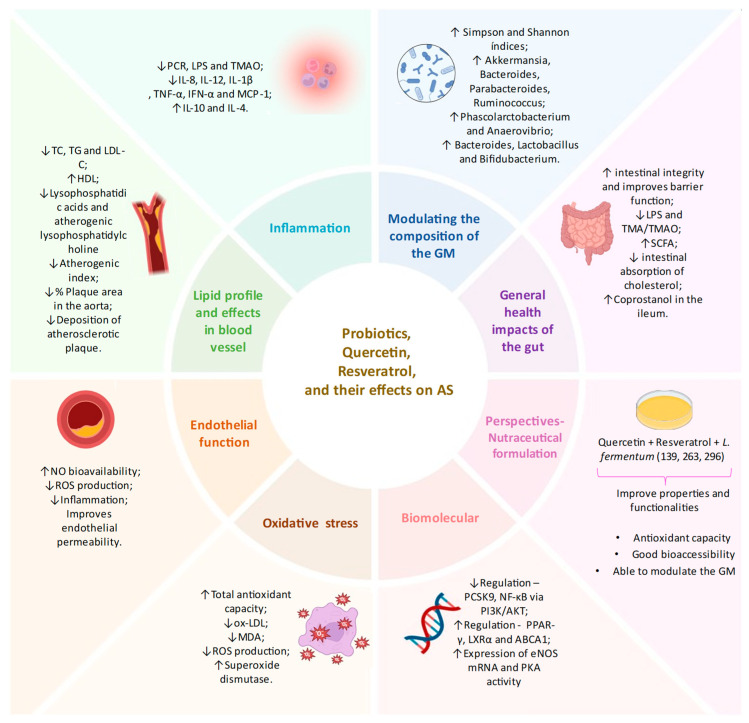
Schematic summarizing the effects of probiotics, quercetin, and resveratrol on atherosclerosis treatment, and presenting future perspectives in the use of nutraceutical formulations combining *Limosilactobacillus fermentum* strains, quercetin, and resveratrol as potential candidates for preclinical studies. ↑ represents an increase. ↓ represents a decrease.

In summary, this section has detailed in vitro functionalities of a novel nutraceutical combining strains of *L. fermentum*, quercetin, and resveratrol. Although these functionalities of the nutraceutical combining strains of *L. fermentum*, quercetin, and resveratrol have only been observed in in vitro assessment, the findings discussed in this study provide scientific evidence to consider the mentioned nutraceutical as a potential candidate for in vivo studies exploring its potential as an adjuvant treatment for AS (Figure 3).

## 9. Conclusions and Future Directions

In conclusion, it has been observed that dysregulated lipid metabolism, oxidative stress, and inflammation are directly involved in the development of AS. In addition, gut dysbiosis may initiate or accelerate the atherogenic process. Furthermore, it was highlighted that probiotic strains, quercetin, and resveratrol may improve atherosclerosis-related parameters when administered individually, possibly due to modulation of the gut microbiota. In general, the main findings of this review pointed out that these compounds can improve the lipid profile, oxidative stress, and inflammation through specific pathways. Curiously, it was observed that the administration of probiotic strains did not significantly change the α-diversity while promoting a distinct gut microbiota composition in the most evaluated studies. On the other hand, quercetin increased α-diversity in evaluated studies. It was also observed that the treatment period for improvement in AS-related parameters was more than 10 weeks, and most studies evaluated the effects of probiotics with a single strain. Perhaps consumption of nutraceutical formulations combining probiotic strains, quercetin, resveratrol, or other phenolic compounds could promote a more diverse gut microbiota profile, alter metabolite production, and reduce intervention time. Therefore, further studies using multi-strain probiotics and phenolic compounds in AS are needed to elucidate whether their effects are superior to those found with a single strain or phenolic compounds.

Headaches, dizziness, nausea, feeling unusually tired or physically weak, digestive system problems such as constipation and diarrhea, muscle pain, sleep problems, and low blood platelet count are common side effects promoted by statin use. Although combined treatment with probiotics and statins is increasing, important clinical evidence shows that the use of probiotics in combination with statins may be essential to treat hyperlipidemia and reduce statin-induced side effects [201,202]. Further clinical trials and meta-analyses are needed to identify the dose, time of intervention, and strains of probiotics that help hyperlipidemia treatment and reduce side effects.

The nutraceutical approach to the treatment of cardiovascular disease is a growing area of investigation. Early clinical trials investigating the effects of nutraceuticals formulated with probiotic strains and red yeast rice extract for 12 weeks have found promising results in improving cardiovascular risk-related parameters, such as the lipid profile and markers of atherosclerosis, such as ox-LDL and apolipoprotein B [203,204]. The combination of *L. fermentum* strains or other probiotics, quercetin, and resveratrol in a nutraceutical formula represents a promising strategy for the prevention or treatment of dyslipidemia and AS. Thus, preclinical and clinical trials will be needed to investigate the effects of this combination on gut microbiota and AS-related parameters.

## Figures and Tables

**Table 1:** Studies using probiotic strains on atherosclerosis treatment through gut microbiota changes.

Treatment	Dose	Model	Time Intervention	Outcomes in Gut Microbiota	References
*Lactiplantibacillus plantarum* ATCC 14917	10^9^ CFU	ApoE^−/−^ mice fed a high-fat diet	12 weeks	↑ intestinal integrity ↑ mRNA expression ZO-1, occluding, claudin-3, MUC-3 ↓ LPS in mesenteric adipose tissue.	Hassan et al., 2024 [78]
Probiotic Mixture (*B*. *breve*, *B*. *longum*, *B*. *infantis*, *L*. *acidophilus*, *L*. *platarum*, *L*. *paracasei*, *L*. *bulgaricus*, *S*. *thermophilus*)	2.78 × 10^11^ CFU/day	ApoE^−/−^ female mice fed a high-fat diet	12 weeks	↔ Distinct compositional profile of the gut microbiota in the ileum, and colon.	Chan et al., 2016 [79]
*L. plantarum* ZDY04	1 × 10^9^ CFU	Female BALB/c mice fed a chow diet with 1.3% choline chloride	4 weeks	↓ serum TMAO ↓ cecal TMA ~ cecal microbiota richness and diversity ↑ *Lachnospiraceae*, *Erysipelotrichaceae*, and *Bacteroidaceae* ↓ *Aerococcaceae* (families) ↑ *Enterorhabdus*, *Succinivibrionaceae* UGG-002, *Lachnospiraceae* UGG-006, *Lachnospiraceae* NK4A136, *Ruminiclostridium* 9, *Lachnospiraceae* XPB1014, *Ruminococcaceae* UCG-014, *Ruminococcaceae* NK4A214, *Christensenellaceae* R-7, and *Rikenellaceae* RC9 (genus) ↔ Distinct microbiota structure	Qiu et al., 2018 [80]
*Lactobacillus mucosae* A1	1 × 10^9^ CFU	ApoE^−/−^ mice fed a high-fat high-cholesterol	13 weeks	~ decreases serum TMA, TMAO, and LBP ~ riches and diversity ↔ Distinct microbiota structure ↓ *Oscillibacter*, *Ruminiclostridium*, *Harryflintia*, *Enterorhabdus*, *Anaerovorax*, *Eubacterium*, *Turicibacter*, *Enterococcus*, unclassified *Ruminococcaceae*, unclassified *Clostridiales*, unclassified *Lachnospiraceae*	Jiang et al., 2020 [81]
*Lactobacillus rhamnosus* GG	1 × 10^7^ CFU once a week	ApoE^−/−^ mice were fed a high-fat diet with an additional *Lactobacillus rhamnosus* GG suspension	12 weeks	~ Chao 1 and Ace indices ↑ Simpson and Shannon indices ↓ Proteobacteria ↑ Firmicutes ↑ *Lactobacillus* ↓ *Desulfovibrionaceae* (genus) ↔ Distinct microbiota diversity	Zhai et al., 2022 [82]
*L*. *plantarum*, *L*. *reuteri*, *L. casei*, *B*. *breve*, and *B*. *adolescentis*	1 × 10^9^ CFU	Female C57BL/6 mice fed a Paigen atherogenic diet	16 weeks	~ α-diversity ↔ Distinct microbiota diversity with an overlap among probiotic intervention groups.	Wang et al., 2022 [77]

The upward arrow represents an increase, the downward arrow represents a decrease, ~ represents no alterations, and ↔ represents distinct composition. *B*. *breve*: *Bifidobacterium breve*, *B*. *longum*: *Bifidobacterium longum*, *B*. *infantis*: *Bifidobacterium infantis*, *L*. *acidophilus*: *Lactobabacillus acidophilus*, *L*. *platarum*: *Lactplantibacillus platarum*, *L*. *paracasei*: *Lacticaseibacillus paracasei*, *L*. *bulgaricus*: *Lactobacillus bulgaricus*, *S*. *thermophilus*: *Streptococcus thermophilus*, *L*. *reuteri*: *Limosilactobacillus reuteri*, *L. casei*: *Lacticaseibacillus casei*, *B*. *adolescentes*: *Bifidobacterium adolescentes*. CFU: colony-forming units. ApoE^−/−^ mice: apolipoprotein E knockout mice. C57BL/6 mice: C57 black 6 mice. BALB/c mice: albino mice. LPS: lipopolysaccharides. mRNA: messenger RNA. ZO-1: Zonula occludens 1. MUC-3: mucin 3A. TMA: trimethylamine. TMAO: trimethylamine N-oxide. LBP: lipopolysaccharide-binding protein.

**Table 2:** Studies using phenolic compounds in atherosclerosis treatment through gut microbiota changes.

Treatment	Dose	Model	Time Intervention	Outcomes in Gut Microbiota	Reference
Quercetin	100 µg/day	LDLR^−/−^ fed a high-fat diet	12 weeks	↑ α-diversity ↑ Akkermansia, Bacteroides, Parabacteroides, and Ruminococcus (phylum)	Nie et al., 2019 [128]
Quercetin	100 mg/kg/day	ApoE^−/−^ mice fed a high-cholesterol diet	12 weeks	↑ *Streptophyta*, *Enterobacter*, *Mobilitalea*, *Clostridium*, *Phascolarctobacterium*, *Candidatus Stoquefichus*, *Faecalimonas*, *Faecalibaculum*, *Anaerovibrio*, *Deltaproteobacteria Unclassified*, and *Acutalibacter* (genus)	Wu et al., 2019 [129]
Quercetin	0.1% *w*/*w* added to diet	ApoE^−/−^ mice fed a high-MAC diet supplemented with quercetin	16 weeks	↑ gut microbiota richness Atherosclerotic plaque areas were negatively associated with the *Eggerthellaceae* and *Erysipelotrichaceae* families and positively associated with the *Lactobacillaceae* family.	Kasahara et al., 2023 [130]
Resveratrol	0.4% added to diet	ApoE^−/−^ mice fed a choline-rich diet supplemented with resveratrol	4 months	↑ *Bacteroides, Akkermansia, Lactobacillus,* and *Bifidobacterium* (genus). ↑ Fecal BA loss.	Chen et al. 2016 [165]
Curcumin	100 mg/kg b.w. by gavage	ApoE^−/−^ mice fed a high-fat diet	2 months	~ α diversity ↑ Firmicute/Bacteroidetes ratio ↑ Abundance of Verrucomicrobia Downregulation *Lactobacillaceae* family ↑ *Unspecifield_S24_7* and *Akkermansia* abundance ↓ *Lactobacillus* abundance ↓ TMAO plasma levels	Zhang; Ou; Chen., 2022 [168]
Curcumin	dosage of 100 mg/kg	LDLr^−/−^ mice fed a high-fat, high-cholesterol Western-type diet	16 weeks	↓ Intestinal permeability ↓ LPS plasma levels ↓ Plasma appearance of FITIC- dextran	Gosh et al., 2014 [174]
Gallic Acid	0.2% gallic acid in drinking water	ApoE^−/−^ mice fed a Paigen atherogenic purified diet	2 weeks	α diversity similar ↓ Ratio of obligate anaerobic/anaerobic bacteria in females ↓ Ratio of gram-positive/negative bacteria in males ↓ Atherosclerotic plaque formation in males	Clark et al., 2022 [169]
Naringin	dosage of 100 mg/kg/day	Female ApoE^−/−^ fed a high-fat diet	16 weeks	↑ Fecal excretion total lipid, total bile acids, and coprostanol ↑ Firmicutes and ↓Bacteroidetes and Verrucomicrobia phylum abundance ↓ *Bacteroides*, *Bifidobacterium*, *Lactococcus* e *Clostridium sensu_stricto_1* ↓ *Streptococcus*, *Desulfovibrio*, *Parasutterella*, and *Bacteroides*	Wang et al., 2020 [170]
Naringin, hesperidin, naringenin, and hesperidin	100 mg/kg/day of each polyphenol	Female ApoE^−/−^ mice fed a high-fat diet	16 weeks	↑ *Lactobacillus*, *Eubacterium coprostanoligenes*, and *Eubacterium brachy* ↓ *Bacteroides*, *Lactococcus*, and *Clostridium_sensu_stricto_1*	Wang et al., 2021 [177]
Procyanidin A2	110 mg/kg body weight/day	Male ApoE^−/−^ fed a high-fat diet	12 weeks	↑ α diversity ↓ Firmicutes/Bacteroidetes ratio ↑ Verrucomicrobia relative abundance ↑ Relative abundance of *Akkermansia*, Prevotellaceae, and *Coriobacteriaceae_UGG-002*	Yang et al., 2021 [171]
Geraniin	80 mg/kg of body weight/day dissolved in drinking water	ApoE^−/−^ mice fed a high-choline diet (0.08% choline diet and 1% choline diet)	12 weeks	↓ TMAO plasma levels ↑ *Bacteroides*, *Alistipes*, *Flavonifractor*, *Clostridium XIVb*, *Butyricicoccus*, *Anaeroplasma*, *Clostridium XI*, *Enterorhabdus*, *Erysipeiotrichaceae_incertae_sedis*, *Anaerotruncus*, *Intestinimonas*, *Vampirovibrio*, and *Butyricimonas* ↓ *Phenomena’s*, *Rhizobium*, *Stenotrophomonas*, *Burkholdeira*, *Serratia*, *Ruminococcus*, *Blautia*, *Parasutterella*, *Pseudomonas*, *Roseburia*, and *Streptophyta*	Lin et al., 2022 [172]
Protocatechuic acid	Western diet supplemented with protocatechuic acid (0.5% or 1.0%)	Male ApoE^−/−^ mice fed a Western diet supplemented with TMAO	12 weeks	↓ TNF-α, MCP-1, IL-1β, and IL-6 plasma levels ↑ α diversity	Ding et al., 2024 [186]

↑ represents an increase. ~ represents no alterations. ↓ represents a decrease. µg: microgram. mg: milligram. % *w*/*w*: weight concentration. MACs: microbiota-accessible carbohydrates. ApoE^−/−^ mice: apolipoprotein E knockout mice. LDLR^−/−^ mice: lipoprotein low-density receptor knockout mice. BA: bile acids. TMAO: trimethylamine N-oxide. LPS: lipopolysaccharide. TNF-α: tumor necrosis factor alpha. MCP-1: monocyte chemotactic protein 1. IL-1β: interleukin 1 beta. IL-6: interleukin 6.

## Data Availability

The original contributions presented in the study are included in the article, further inquiries can be directed to the corresponding author.

## Abbreviations

CVD: cardiovascular disease; AS: atherosclerosis; LDL: low-density lipoprotein; TMAO: trimethylamine N-oxide; LPS: lipopolysaccharide; VSCM: vascular smooth muscle cells; Ox-LDL: oxidized LDL; PUFAs: polyunsaturated fatty acids; VCAM-1: vascular cell adhesion molecule 1; E-selectins: endothelium selectins; P-selectins: platelet selectins; ROS: reactive oxygen species; NADPH oxidase: nicotinamide adenine dinucleotide phosphate oxidase; eNOS: uncoupled endothelial NO synthase; NF-kB: nuclear factor kappa B; MMP: metalloproteinase; SCFAs: short-chain fatty acids; TJs: tight junctions; TMA: trimethylamine; FMT: fecal microbiota transplantation; TRL4: Toll-like receptor 4; TNF-α: tumor necrosis factor alpha; IL: interleukin; HDL: high-density lipoprotein; VLDL: very low-density lipoprotein; NO: nitric oxide; ROS: reactive oxygen species; PCSK9: proprotein convertase subtilisin/kexin type 9; CD36: cluster of differentiation 36; PPARγ: peroxisome proliferator-activated receptor γ; LXRα: liver X receptor α; ABCA1: ATP-binding cassette transporter A1; PI3K: phosphatidylinositide 3 kinase; AKT: protein kinase B; HUVECs: human umbilical vein endothelial cells; ICAM-1: intercellular adhesion molecule 1; TG: triglyceride; low-MAC: low-fat-, low-microbiota-accessible carbohydrate diet; high-MAC: standard grain-based chow diet; TC: total cholesterol; HMG-CoA: 3-hydroxy-3-methylglutaryl coenzyme A; CYP7A1: cholesterol 7α-hydroxylase; MDA: malondialdehyde; GLP-1: glucagon-like peptide 1; BSH: bile salt hydrolase; BA: bile acid; FXR-FGF15: nuclear farnesoid X receptor and fibroblast growth factor 15; LBP: lipopolysaccharide-binding protein; ASVs: all-amplicon sequence variants; SOD: superoxide dismutase; ApoE^−/−^: apolipoprotein E knockout mice; LDLr^−/−^: LDL receptor knockout animal; Cd: cadmium.
